# Substance use patterns and unprotected sex among street-involved youth in a Canadian setting: a prospective cohort study

**DOI:** 10.1186/s12889-015-2627-z

**Published:** 2016-01-05

**Authors:** Tessa Cheng, Caitlin Johnston, Thomas Kerr, Paul Nguyen, Evan Wood, Kora DeBeck

**Affiliations:** 1 British Columbia Centre for Excellence in HIV/AIDS, 608-1081 Burrard Street, Vancouver, B.C. Canada V6Z 1Y6; 2 Faculty of Health Sciences, Simon Fraser University, Blusson Hall, Room 11300, 8888 University Drive, Burnaby, B.C. Canada V5A 1S6; 3 BC Women Hospital and Health Centre, 4500 Oak St, Vancouver, B.C. Canada V6H 3V5; 4 Faculty of Medicine, University of British Columbia, 317 - 2194 Health Sciences Mall, Vancouver, B.C. Canada V6T 1Z3; 5 School of Public Policy, Simon Fraser University, 515 West Hastings Street, Suite 3271, Vancouver, B.C. Canada V6B 5K3

**Keywords:** Street-youth, Unprotected sex, Addictions, Risk behaviour

## Abstract

**Background:**

Rates of sexually transmitted infections (STI) and unplanned pregnancy are high among youth. While the intersection between drug and alcohol use and unprotected sex is well recognized, few studies have examined the relationship between substance use patterns and unprotected sex among high risk-populations such as street-involved youth.

**Methods:**

Data were derived from the At-Risk Youth Study (ARYS), a prospective cohort of street-involved youth from Vancouver, Canada. Generalized estimating equations (GEE) were used to examine substance use patterns that were independently associated with unprotected sex, defined as (vaginal or anal) sexual intercourse without consistent condom use.

**Results:**

Between September 2005 and May 2013, 1,026 youth were recruited into the ARYS cohort and 75 % (*n* = 766) reported engaging in recent unprotected sex at some point during the study period. In a multivariable analysis, female gender (adjusted odds ratio [AOR] = 1.46, 95 % confidence interval [CI]: 1.18-1.81), Caucasian ancestry (AOR = 1.38, 95 % CI: 1.13-1.68), being in a stable relationship (AOR = 4.64, 95 % CI: 3.82-5.65), having multiple sex partners (AOR = 2.60, 95 % CI: 2.18-3.10) and the following substance use patterns were all independently associated with recent unprotected sex: injection or non-injection crystal methamphetamine use (AOR = 1.21, 95 % CI: 1.03-1.43), injection or non-injection cocaine use (AOR = 1.20, 95 % CI: 1.02-1.41), marijuana use (AOR = 1.23, 95 % CI: 1.02-1.49), ecstasy use (AOR = 1.23, 95 % CI: 1.01-1.48) and alcohol use (AOR = 1.31, 95 % CI: 1.11-1.55) (all *p* < 0.05).

**Conclusions:**

Unprotected sex was prevalent among street-involved youth in this setting, and independently associated with female gender and a wide range of substance use patterns. Evidence-based and gender-informed sexual health interventions are needed in addition to increased access to youth-centered addiction treatment services. STI testing and linkages to healthcare professionals remain important priorities for street-involved youth, and should be integrated across all health and social services.

## Background

Youth are at a critical stage of development as they initiate sexual and substance use behaviours that shape their health throughout adulthood [1]. The evidence suggests, however, that this crucial transition period is often overlooked and not adequately addressed by healthcare providers in many settings [2]. This is especially important for street-involved youth, who commonly experience trauma and abuse before entering street life [3], and adverse childhood events have been linked with an increased risk of illicit drug use [4] and, among women, sexual risk taking [5].

Despite efforts to increase safer sex practices among youth [6], in 2008 nearly half of all new sexually transmitted infections (STI) in the United States occurred among those aged 15–24 [7]. Although condom use among Canadian youth has been estimated to be over 60 % since 2003 [8], the rate of condom use among Canadian street youth is estimated to hover around 50 % [9]. The prevalence of chlamydia and gonorrhoea has also been found to be disproportionately higher among street-involved youth [9], with female youth generally having higher STI infection rates than males [9–11]. These differences in condom use and STI infection highlight the increased vulnerability of street-involved youth, and indicate that street-involved youth continue to experience disproportionate negative health outcomes and barriers to condom use.

The relationship between substance use and sexual activity in the general population is well-established, as previous studies have linked alcohol and illicit drug use with high-risk sexual behaviours such as increased frequency of intercourse, multiple sexual partners, and lower rates of condom use [12–15]. Despite increasing recognition of higher rates of STI among street-involved youth, less is known about substance use patterns and unprotected sex among street-involved youth who navigate a complex risk environment of danger on a daily basis [16]. Given that few prospective longitudinal studies have examined unprotected sex and associated drug-related risk factors among this population, the present study was conducted to examine whether use of specific substances were associated with engaging in unprotected sex among street-involved youth.

## Methods

Street-involved youth in Vancouver, Canada were recruited into a prospective cohort known as the At-Risk Youth Study (ARYS), which has previously been described in detail [17]. Briefly, persons were eligible if they had used illicit drugs other than marijuana in the past 30 days, were between the ages 14 and 26, provided informed consent, and were ‘street-involved’ (defined as being temporarily or absolutely without housing in the preceding six months, or having accessed street-based youth services during that time). Participants who were unable to provide informed consent at the time due to intoxication, mental health issues, or inability to communicate in English were not enrolled into our study. At baseline and semi-annually, participants complete an interviewer-administered questionnaire and receive a stipend ($20 CDN) at each study visit. The Providence Health Care/University of British Columbia Research Ethics Board approved the study. Based on their street-involved status, youth under the age of 19 were considered emancipated minors and, consistent with provincial law allowing emancipated minors to consent to participate in research on their own behalf, were permitted to participate without parental consent.

This study included all participants who attended a study visit between September 2005 and May 2013. All participants were asked about their engagement in sexual activity over the last six months. For both same and opposite sex partnerships, participants were also asked to report how often a condom was used during vaginal and/or anal intercourse in the last six months. Possible responses included: always, usually, sometimes, occasionally, and never. In line with previous studies of condom use among street-involved youth [18], unprotected sex (yes vs. no) was defined based on reports of sexual activity and condom use. Specifically, unprotected sex was defined as reporting any insertive or receptive sex and “inconsistent” (i.e., usually, sometimes, occasionally, or never) condom use. No unprotected sex was defined as “always” reporting condom use during sexual encounters or reporting no sexual activity.

Explanatory variables of interest included the following socio-demographic information: female gender (yes vs. no); age (≥median age vs. <median age); ethnicity (Caucasian vs. other); currently being in a stable relationship, defined as being legally married, or common law, or having a regular partner (yes vs. no); and homelessness, defined as having no fixed address, sleeping on the street, couch surfing, or staying in a shelter or hostel (yes vs. no). Substance use variables included: any injection or non-injection use of crystal methamphetamine (yes vs. no); any injection or non-injection use of powder cocaine (yes vs. no); any injection or non-injection use of heroin (yes vs. no); any injection or non-injection use of crack cocaine (yes vs. no); any marijuana use (yes vs. no); any non-injection ecstasy use (yes vs. no); any alcohol use, defined as drinking beer, cider, coolers, wine, liquor, or other sources of alcohol (yes vs. no); binge drug use, defined as a period of using injection or non-injection drugs more often than usual based on participant responses to the following question: “In the past six months, did you go on runs or binges (that is, when you used non-injection drugs/injected drugs more than usual)” (yes vs. no); and any injection drug use (yes vs. no). Other risk characteristics included: multiple concurrent sexual partners (excluding those from sex work), based on responses to the following question: “In the last 6 months, how many different women/men have you had oral, vaginal or anal sex with, excluding those with whom you had sex in exchange for money or something else?” (>1 vs. ≤1); and engaging in sex work, defined as exchanging sex for money, shelter, drugs or other commodities (yes vs. no). Unless otherwise stated, all behavioural and risk variables refer to activities in the past six months.

First, we examined baseline characteristics from participants’ first study visit, stratified by unprotected sex, using Pearson’s χ^2^ test. Second, we examined reports of unprotected sex in the past six months during study follow-up using generalized estimating equations (GEE) with a logit link function and an exchangeable correlation structure for the analysis of correlated data [19]. Bivariate GEE analyses were used to determine factors associated with unprotected sex. In order to adjust for potential confounding in the multivariable GEE analysis, variables significant at the *p* < 0.10 threshold in bivariate analyses were used in the backwards model selection process. The model with the best overall fit was determined by the lowest quasilikelihood under the independence model criterion (QIC) value [20]. All statistical analyses were performed using the SAS software version 9.3 (SAS Institute, Cary, NC), and all *p*-values are two sided.

### Availability of data and materials

The data from this study are not available in a public repository due to ethical concerns. Participants were assured during the informed consent process and throughout each study visit that their responses were confidential.

## Results

Between September 2005 and May 2013, 1,026 ARYS youths were eligible for this analysis. The median age at baseline was 21 (inter-quartile range [IQR]: 19-23), 327 (32 %) were female, and 698 (68 %) identified as Caucasian. Of this sample, 590 (58 %) youths reported engaging in unprotected sex at baseline, with an additional 176 (17 %) youths engaging in unprotected sex during follow-up. A total of 75 % of study participants reported unprotected sex over the study period. Participants contributed 3,605 observations during the study period, which included 1,903 (53 %) reports of unprotected sex. The median number of study visits was 3 (IQR: 1-5). Baseline descriptive frequencies and bivariate analyses of characteristics of this study sample, stratified by reports of unprotected sex at baseline, are displayed in Table 1.

**Table 1:** Baseline characteristics^a^ of street-involved youth in Vancouver stratified by unprotected sex in L6M,^b^ 2005-2013 (*n* = 1,026)

Characteristic	Total (%) (*n* = 1,026)	Unprotected Sex in L6M^b^		Odds Ratio (95 % CI)
		Yes (%) (*n* = 590)	No (%) (*n* = 436)	
Female gender				
(yes vs. no)	327 (31.87)	213 (36.10)	114 (26.15)	1.60 (1.22-2.09)**
Age				
(≥median vs. <median)	624 (60.82)	354 (60.00)	270 (61.93)	0.92 (0.72-1.19)
Caucasian ancestry				
(yes vs. no)	698 (68.03)	420 (71.19)	278 (63.76)	1.40 (1.08-1.83)*
Stable relationship				
(yes vs. no)	296 (28.85)	224 (37.97)	72 (16.51)	3.16 (2.34-4.28)***
Homelessness in L6M^b^				
(yes vs. no)	752 (73.29)	451 (76.44)	301 (69.04)	1.49 (1.12-1.97)*
Any crystal meth use in L6M^b,c^				
(yes vs. no)	461 (44.93)	276 (46.78)	185 (42.43)	1.17 (0.91-1.50)
Any cocaine use in L6M^b,c^				
(yes vs. no)	506 (49.32)	312 (52.88)	194 (44.50)	1.38 (1.07-1.77)*
Any heroin use in L6M^b,c^				
(yes vs. no)	358 (34.89)	198 (33.56)	160 (36.70)	0.87 (0.67-1.13)
Any crack use in L6M^b,c^				
(yes vs. no)	611 (59.55)	357 (60.51)	254 (58.26)	1.08 (0.84-1.40)
Any marijuana use in L6M^b^				
(yes vs. no)	903 (88.01)	516 (87.46)	387 (88.76)	0.86 (0.58-1.27)
Any ecstasy use in L6M^b^				
(yes vs. no)	334 (32.55)	211 (35.76)	123 (28.21)	1.42 (1.09-1.86)*
Any alcohol use in L6M^b^				
(yes vs. no)	833 (81.19)	499 (84.58)	334 (76.61)	1.66 (1.21-2.28)**
Binge drug use in L6M^b,c^				
(yes vs. no)	433 (42.20)	265 (44.92)	168 (38.53)	1.30 (1.01-1.67)*
Injection drug use in L6M^b^				
(yes vs. no)	306 (29.82)	173 (29.32)	133 (30.50)	0.94 (0.72-1.23)
Multiple sex partners in L6M^b^				
(>1 vs. ≤1)	558 (54.39)	371 (62.88)	187 (42.89)	2.26 (1.75-2.91)***
Sex work in L6M^b^				
(yes vs. no)	108 (10.53)	66 (11.19)	42 (9.63)	1.18 (0.79-1.78)

Notes:

^a^Characteristics for all participants were measured from the first study visit

^b^‘L6M’ refers to behaviours and activities occurring in the last six months

^c^Refers to injection or non-injection use

* significant at *p* < 0.05; ** significant at *p* < 0.005; *** significant at *p* < 0.001

The bivariate and multivariable GEE analyses of the socio-demographic, drug use, and risk factors that were associated with unprotected sex are displayed in Table 2. In the multivariable GEE analysis, factors that were positively and independently associated with having unprotected sex (*p* < 0.05) included: female gender (adjusted odds ratio [AOR] = 1.46, 95 % confidence interval [CI]: 1.18-1.81), Caucasian ancestry (AOR = 1.38, 95 % CI: 1.13-1.68), being in a stable relationship (AOR = 4.64, 95 % CI: 3.82-5.65), any injection or non-injection crystal methamphetamine use (AOR = 1.21, 95 % CI: 1.03-1.43), any injection or non-injection cocaine use (AOR = 1.20, 95 % CI: 1.02-1.41), any marijuana use (AOR = 1.23, 95 % CI: 1.02-1.49), any non-injection ecstasy use (AOR = 1.23, 95 % CI: 1.01-1.48), any alcohol use (AOR = 1.31, 95 % CI: 1.11-1.55), and having multiple sex partners (AOR = 2.60, 95 % CI: 2.18-3.10).

**Table 2:** Bivariate and multivariable GEE analyses of factors associated with unprotected sex in L6M^a^ (*n* = 1,026)

Characteristic	Unadjusted	Adjusted
	Odds Ratio (95 % CI)	Odds Ratio (95 % CI)
Female gender		
(yes vs. no)	1.54 (1.25-1.90)***	1.46 (1.18-1.81)**
Age		
(≥median vs. <median)	0.78 (0.64-0.95)*	
Caucasian ancestry		
(yes vs. no)	1.32 (1.08-1.61)*	1.38 (1.13-1.68)**
Stable relationship		
(yes vs. no)	3.11 (2.63-3.68)***	4.64 (3.82-5.65)***
Homelessness in L6M^a^		
(yes vs. no)	1.16 (1.02-1.32)*	1.15 (0.99-1.33)
Any crystal meth use in L6M^a,b^		
(yes vs. no)	1.20 (1.03-1.40)*	1.21 (1.03-1.43)*
Any cocaine use in L6M^a,b^		
(yes vs. no)	1.43 (1.24-1.64)***	1.20 (1.02-1.41)*
Any heroin use in L6M^a,b^		
(yes vs. no)	0.90 (0.77-1.05)	
Any crack use in L6M^a,b^		
(yes vs. no)	1.07 (0.94-1.23)	
Any marijuana use in L6M^a^		
(yes vs. no)	1.37 (1.17-1.60)***	1.23 (1.02-1.49)*
Any ecstasy use in L6M^a^		
(yes vs. no)	1.41 (1.19-1.67)***	1.23 (1.01-1.48)*
Any alcohol use in L6M^a^		
(yes vs. no)	1.45 (1.26-1.68)***	1.31 (1.11-1.55)**
Binge drug use in L6M^a,b^		
(yes vs. no)	1.13 (0.99-1.30)	
Injection drug use in L6M^a^		
(yes vs. no)	1.00 (0.85-1.16)	
Multiple sex partners in L6M^a^		
(>1 vs. ≤ 1)	1.86 (1.60-2.17)***	2.60 (2.18-3.10)***
Sex work in L6M^a^		
(yes vs. no)	1.19 (0.92-1.55)	

Notes:

^a^‘L6M’ refers to behaviours and activities occurring in the last six months

^b^Refers to injection or non-injection use

*significant at *p* < 0.05; ** significant at *p* < 0.005; *** significant at *p* < 0.001

## Discussion

In the present study, 766 (75 %) youth reported recently engaging in unprotected sex during the study period and the majority of study observations included a report of recent unprotected sex. Female gender, Caucasian ancestry, substance use, monogamous relationships, and having multiple concurrent sex partners, were independently and positively associated with unprotected sex. The high prevalence of unprotected sex in this study aligns with previous findings that up to 25 % of street-youth have never used condoms and 56 % did not use condoms the last time they had sex under the influence of substances [10, 21]; this contrasts with a much higher proportion of condom use at last intercourse among the general youth population aged 20-24 in 2009/2010 (63 %) [8]. It is unclear, however, if our study outcome of “any unprotected sex in the recent six months” is comparable to “unprotected sex at last sexual intercourse”.

A number of different drugs were positively and significantly associated with unprotected sex in our study. Experimentation with alcohol at an early age is common among young people [22], and this study found that youth who reported alcohol use were more likely to report having unprotected sex. Alcohol is known to lower inhibitions which increases the likelihood of engaging in sexual activities that one might not normally partake in, such as sexual encounters with strangers, anal intercourse, and sex without a condom [23–25].

The null findings for binge drug use and injection drug use in the current analysis indicate that youth in our sample who engage in unprotected sex are not more likely to engage in especially risky drug use patterns. However, crystal methamphetamine and cocaine use were significantly associated with engaging in unprotected sex in our analysis. This is consistent with previous research findings that stimulant drug use heightens sexual arousal and lowers inhibitions, resulting in a higher likelihood of engaging in risky sexual behavior [26, 27]. The link between stimulant drug use, increased sexual arousal and reduced inhibitions, resulting in lower condom use, is particularly well documented in the context of sexual health among men who have sex with men [28]. The effect of stimulant use has also been found to increase risk of STI transmission among adults who use illicit drugs, in part by facilitating longer periods of sexual activity which can lead to increased risk of condom breakage [27, 29].

Our results provide further evidence that reducing stimulant drug use may prevent high levels of unprotected sex among this population. It is therefore of concern that vulnerable youth report high rates of difficulty accessing addiction treatment [30, 31]. Sustained efforts to improve engagement and retention in addiction treatment are warranted and can be expected to have positive health benefits beyond reductions in substance use [32, 33]. For youth who are unable or unwilling to reduce engagement in stimulant drug use, alternative interventions are needed. There is some evidence to suggest that low-threshold services such as supervised injection facilities [34] and needle exchange programs [35] may increase condom use; however, more studies to assess whether these secondary benefits would be realized with street-involved youth are needed. In addition, research indicates that the risks of HIV transmission through sexual intercourse can be reduced through expanded HIV testing and treatment [36]. Consequently, STI and HIV testing for vulnerable youth who use stimulants should be a public health priority and integrated into all healthcare services.

It is noteworthy that ecstasy and marijuana use were each also positively and significantly associated with unprotected sex in this study. Ecstasy is known to induce feelings of euphoria, friendliness, and enhanced sensuality [37], and previous research has linked ecstasy use [38, 39] and marijuana use [40, 41] with sexual risk-taking. However, studies in this area have not been consistent and further investigation into the association between ecstasy and risky sexual behavior including inconsistent condom use is warranted [42]. Similarly, the null findings for crack cocaine use and inconsistent condom use in the current study contrast with research in other settings among drug-using youth [43], suggesting more investigation is needed.

Study findings also show that female youth are significantly more likely to engage in unprotected sex, which is linked with complex interactions of gender inequality, power, and socio-structural context [44]. Our results indicate that condoms are inconsistently used among participants in stable relationships and who have multiple concurrent sex partners (38 % and 63 % at baseline, respectively). The positive relationship between stable relationships and inconsistent condom use aligns with previous research [45, 46], however, the association between multiple concurrent sex partners and inconsistent condom use is novel. These results point to the need for tailored gender-informed interventions to support consistent condom use among sexually active street-involved youth [47, 48].

There are a number of study limitations. The absence of a probability sample limits the ability of this study to generalize to other settings, although our extensive recruitment efforts resulted in a similar sample to those found in other studies of Vancouver street-involved youth [49, 50]. Self-report surveys are also vulnerable to recall and socially-desirable response biases [51]; however, under-reporting of illicit drug use and sexual practices are expected to bias our results to the null.

## Conclusions

This study demonstrates that unprotected sex remains highly prevalent among drug-using youth in this setting and a number of illicit drugs were independently associated with inconsistent condom use. Findings suggest that improving access to evidence-based and youth-centered addiction treatment to reduce problematic substance use can be expected to also prevent risky sexual behaviour [52]. For those who continue to engage in substance use, better connections to healthcare services and STI testing are needed across the continuum of care. The heightened risk of unprotected sex among female youth in this study also highlights the need for gender-informed interventions to support consistent condom use among street-involved youth.

## Abbreviations

ARYS: At-Risk Youth Study
AOR: Adjusted Odds Ratio
CI: Confidence Interval
GEE: Generalized Estimating Equation
IQR: Inter-Quartile Range
STI: Sexually Transmitted Infection
